# The Polynomial Complexity of Vector Addition Systems with States

**DOI:** 10.1007/978-3-030-45231-5_32

**Published:** 2020-04-17

**Authors:** Florian Zuleger

**Affiliations:** 8 ENS/CNRS, Université Paris-Saclay, Cachan, France; 9 University of Duisburg-Essen, Duisburg, Germany; TU Wien, Vienna, Austria

## Abstract

Vector addition systems are an important model in theoretical computer science and have been used in a variety of areas. In this paper, we consider vector addition systems with states over a parameterized initial configuration. For these systems, we are interested in the standard notion of computational time complexity, i.e., we want to understand the length of the longest trace for a fixed vector addition system with states depending on the size of the initial configuration. We show that the asymptotic complexity of a given vector addition system with states is either $\varTheta (N^k)$ for some computable integer *k*, where *N* is the size of the initial configuration, or at least exponential. We further show that *k* can be computed in polynomial time in the size of the considered vector addition system. Finally, we show that $1 \le k \le 2^n$, where *n* is the dimension of the considered vector addition system.

## 1 Introduction

Vector addition systems (VASs) [13], which are equivalent to Petri nets, are a popular model for the analysis of parallel processes [7]. Vector addition systems with states (VASSs) [10] are an extension of VASs with a finite control and are a popular model for the analysis of concurrent systems, because the finite control can for example be used to model shared global memory [12]. In this paper, we consider VASSs over a parameterized initial configuration. For these systems, we are interested in the standard notion of computational time complexity, i.e., we want to understand the length of the longest execution for a fixed VASS depending on the size of the initial configuration. VASSs over a parameterized initial configuration naturally arise in two areas: 1) *The parameterized verification problem.* For concurrent systems the number of system processes is often not known in advance, and thus the system is designed such that a template process can be instantiated an arbitrary number of times. The problem of analyzing the concurrent system for all possible system sizes is a common theme in the literature [9, 8, 1, 11, 4, 2, 3]. 2) *Automated complexity analysis of programs.* VASSs (and generalizations) have been used as backend in program analysis tools for automated complexity analysis [18–20]. The VASS considered by these tools are naturally parameterized over the initial configuration, modelling the dependency of the program complexity on the program input. The cited papers have proposed practical techniques but did not give complete algorithms.

Two recent papers have considered the computational time complexity of VASSs over a parameterized initial configuration. [15] presents a PTIME procedure for deciding whether a VASS is polynomial or at least exponential, but does not give a precise analysis in case of polynomial complexity. [5] establishes the precise asymptotic complexity for the special case of VASSs whose configurations are linearly bounded in the size of the initial configuration. In this paper, we generalize both results and fully characterize the asymptotic behaviour of VASSs with polynomial complexity: We show that the asymptotic complexity of a given VASS is either $\Theta(N^k)$ for some computable integer $k$, where $N$ is the size of the initial configuration, or at least exponential. We further show that $k$ can be computed in PTIME in the size of the considered VASS. Finally, we show that $1 \leq k \leq 2^n$, where $n$ is the dimension of the considered VASS.

### 1.1 Overview and Illustration of Results

We discuss our approach on the VASS $\mathcal{V}_{run}$, stated in Figure 1, which will serve as running example. The VASS has dimension 3 (i.e., the vectors annotating the transitions have dimension 3) and four states $s_1, s_2, s_3, s_4$. In this paper we will always represent vectors using a set of variables $Var$, whose cardinality equals the dimension of the VASS. For $\mathcal{V}_{run}$ we choose $Var = \{x, y, z\}$ and use $x, y, z$ as indices for the first, second and third component of 3-dimensional vectors. The configurations of a VASS are pairs of states and valuations of the variables to non-negative integers. A step of a VASS moves along a transition from the current state to a successor state, and adds the vector labelling the transition to the current valuation; a step can only be taken if the resulting valuation is non-negative. For the computational time complexity analysis of VASSs, we consider traces (sequences of steps) whose initial configurations consist of a valuation whose maximal value is bounded by $N$ (the parameter used for bounding the size of the initial configuration). The computational time complexity is then the length of the longest trace whose initial configuration is bounded by $N$. For ease of exposition, we will in this paper only consider VASSs whose control-flow graph is *connected*. (For the general case, we remark that one needs to decompose a VASS into its strongly-connected components (SCCs), which can then be analyzed in isolation, following the DAG-order of the SCC decomposition; for this, one slightly needs to generalize the analysis in this paper to initial configurations with values $\Theta(N^{k_x})$ for every variable $x \in Var$, where $k_x \in \mathbb{Z}$.) For ease of exposition, we further consider traces over arbitrary initial states (instead of some fixed initial state); this is justified because for a fixed initial state one can always restrict the control-flow graph to the reachable states, and then the two options result in the same notion of computational complexity (up to a constant offset, which is not relevant for our asymptotic analysis).

In order to analyze the computational time complexity of a considered VASS, our approach computes *variable bounds* and *transition bounds*. A variable bound is the maximal value of a variable reachable by any trace whose initial configuration is bounded by $N$. A transition bound is the maximal number of times a transition appears in any trace whose initial configuration is bounded by $N$. For

$\mathcal{V}_{run}$, our approach establishes the linear variable bound $\Theta(N)$ for $x$ and $y$, and the quadratic bound $\Theta(N^2)$ for $z$. We note that because the variable bound of $z$ is quadratic and not linear, $\mathcal{V}_{run}$ cannot be analyzed by the procedure of [5]. Our approach establishes the bound $\Theta(N)$ for the transitions $s_1 \to s_3$ and $s_4 \to s_2$, the bound $\Theta(N^2)$ for transitions $s_1 \to s_2$, $s_2 \to s_1$, $s_3 \to s_4$, $s_4 \to s_3$, and the bound $\Theta(N^3)$ for all self-loops. The computational complexity of $\mathcal{V}_{run}$ is then the maximum of all transition bounds, i.e., $\Theta(N^3)$. In general, our main algorithm (Algorithm 1 presented in Section 4) either establishes that the VASS under analysis has at least exponential complexity or computes asymptotically precise variable and transition bounds $\Theta(N^k)$, with $k$ computable in PTIME and $1 \leq k \leq 2^n$, where $n$ is the dimension of the considered VASS. We note that our upper bound $2^n$ also improves the analysis of [15], which reports an exponential dependence on the number of transitions (and not only on the dimension).

We further state a family $\mathcal{V}_n$ of VASSs, which illustrate that $k$ can indeed be exponential in the dimension (the example can be skipped on first reading). $\mathcal{V}_n$ uses variables $x_{i,j}$ and consists of states $s_{i,j}$, for $1 \leq i \leq n$ and $j = 1, 2$. We note that $\mathcal{V}_n$ has dimension $2n$. $\mathcal{V}_n$ consists of the transitions

- $s_{i,1} \xrightarrow{d} s_{i,2}$, for $1 \leq i \leq n$, with $d(x_{i,1}) = -1$ and $d(x) = 0$ for all $x \neq x_{i,1}$,
- $s_{i,2} \xrightarrow{d} s_{i,1}$, for $1 \leq i \leq n$, with $d(x) = 0$ for all $x$,
- $s_{i,1} \xrightarrow{d} s_{i,1}$, for $1 \leq i \leq n$, with $d(x_{i,1}) = -1$, $d(x_{i,2}) = 1$, $d(x_{i+1,1}) = d(x_{i+1,2}) = 1$ in case $i < n$, and $d(x) = 0$ for all other $x$,
- $s_{i,2} \xrightarrow{d} s_{i,2}$, for $1 \leq i \leq n$, with $d(x_{i,1}) = 1$, $d(x_{i,2}) = -1$, and $d(x) = 0$ for all other $x$,
- $s_{i,1} \xrightarrow{d} s_{i+1,1}$, for $1 \leq i < n$, with $d(x_{i,1}) = -1$ and $d(x) = 0$ for all $x \neq x_{i,1}$,
- $s_{i+1,2} \xrightarrow{d} s_{i,2}$, for $1 \leq i < n$, with $d(x) = 0$ for all $x$.

$\mathcal{V}_{exp}$ in Figure 1 depicts $\mathcal{V}_n$ for $n = 3$, where the vector components are stated in the order $x_{1,1}, x_{1,2}, x_{2,1}, x_{2,2}, x_{3,1}, x_{3,2}$. It is not hard to verify for all $1 \leq i \leq n$ that $\Theta(N^{2^{i-1}})$ is the precise asymptotic variable bound for $x_{i,1}$ and $x_{i,2}$, that $s_{i,1} \to s_{i,2}$, $s_{i,2} \to s_{i,1}$ and $s_{i,1} \to s_{i+1,1}$, $s_{i+1,2} \to s_{i,2}$ in case $i < n$, and that $\Theta(N^{2^i})$ is the precise asymptotic transition bound for $s_{i,1} \to s_{i,1}$, $s_{i,2} \to s_{i,2}$ (Algorithm 1 can be used to find these bounds).

### 1.2 Related Work

A celebrated result on VASs is the EXPSPACE-completeness [16, 17] of the boundedness problem. Deciding termination for a VAS with a *fixed* initial configuration can be reduced to the boundedness problem, and is therefore also EXPSPACE-complete; this also applies to VASSs, whose termination problem can be reduced to the VAS termination problem. In contrast, deciding the termination of VASSs for *all* initial configurations is in PTIME. It is not hard to see that non-termination over all initial configurations is equivalent to the existence of non-negative cycles (e.g., using Dickson's Lemma [6]). Kosaraju and Sullivan have given a PTIME procedure for the detection of zero-cycles [14], which can be easily be adapted to non-negative cycles. The existence of zero-cycles is decided

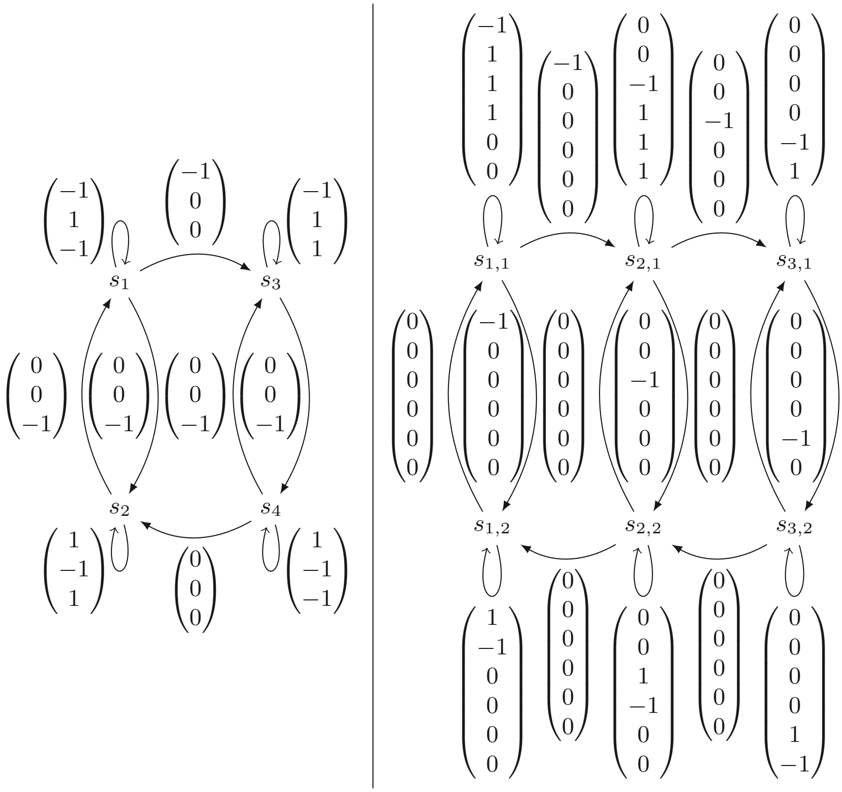

**Fig. 1.** VASS $\mathcal{V}_{run}$ (left) and VASS $\mathcal{V}_{exp}$ (right)

by the repeated use of a constraint system in order to remove transitions that can definitely not be part of a zero-cycle. The algorithm of Kosaraju and Sullivan forms the basis for both cited papers [15, 5], as well as the present paper.

A line of work [18–20] has used VASSs (and their generalizations) as backends for the automated complexity analysis of C programs. These algorithms have been designed for practical applicability, but are not complete and no theoretical analysis of their precision has been given. We point out, however, that these papers have inspired the Bound Proof Principle in Section 5.

## 2 Preliminaries

*Basic Notation.* For a set $X$ we denote by $|X|$ the number of elements of $X$. Let $\mathbb{S}$ be either $\mathbb{N}$ or $\mathbb{Z}$. We write $\mathbb{S}^I$ for the set of vectors over $\mathbb{S}$ indexed by some set $I$. We write $\mathbb{S}^{I\times J}$ for the set of matrices over $\mathbb{S}$ indexed by $I$ and $J$. We write $\mathbf{1}$ for the vector which has entry 1 in every component. Given $a \in \mathbb{S}^I$, we write $a(i) \in \mathbb{S}$ for the entry at line $i \in I$ of $a$, and $\|a\| = \max_{i\in I} |a(i)|$ for the maximum absolute value of $a$. Given $a \in \mathbb{S}^I$ and $J \subseteq I$, we denote by $a|_J \in \mathbb{S}^J$ the restriction of $a$ to $J$, i.e., we set $a|_J(i) = a(i)$ for all $i \in J$. Given $A \in \mathbb{S}^{I\times J}$, we write $A(j)$ for the vector in column $j \in J$ of $A$ and $A(i,j) \in \mathbb{S}$ for the entry in column $i \in I$ and row $j \in J$ of $A$. Given $A \in \mathbb{S}^{I \times J}$ and $K \subseteq J$, we denote by $A|_K \in \mathbb{S}^{I \times K}$ the restriction of $A$ to $K$, i.e., we set $A|_K(i,j) = A(i,j)$ for all $(i,j) \in I \times K$. We write **Id** for the square matrix which has entries 1 on the diagonal and 0 otherwise. Given $a, b \in \mathbb{S}^I$ we write $a+b \in \mathbb{S}^I$ for component-wise addition, $c \cdot a \in \mathbb{S}^I$ for multiplying every component of $a$ by some $c \in \mathbb{S}$ and $a \geq b$ for component-wise comparison. Given $A \in \mathbb{S}^{I \times J}$, $B \in \mathbb{S}^{J \times K}$ and $x \in \mathbb{S}^J$, we write $AB \in \mathbb{S}^{I \times K}$ for the standard matrix multiplication, $Ax \in \mathbb{S}^I$ for the standard matrix-vector multiplication, $A^T \in \mathbb{S}^{J \times I}$ for the transposed matrix of $A$ and $x^T \in \mathbb{S}^{1 \times J}$ for the transposed vector of $x$.

*Vector Addition System with States (VASS).* Let $\mathit{Var}$ be a finite set of variables. A vector addition system with states (VASS) $\mathcal{V} = (\mathit{St}(\mathcal{V}), \mathit{Trns}(\mathcal{V}))$ consists of a finite set of *states* $\mathit{St}(\mathcal{V})$ and a finite set of *transitions* $\mathit{Trns}(\mathcal{V})$, where $\mathit{Trns}(\mathcal{V}) \subseteq \mathit{St}(\mathcal{V}) \times \mathbb{Z}^{\mathit{Var}} \times \mathit{St}(\mathcal{V})$; we call $n = |\mathit{Var}|$ the *dimension* of $\mathcal{V}$. We write $s_1 \xrightarrow{d} s_2$ to denote a transition $(s_1, d, s_2) \in \mathit{Trns}(\mathcal{V})$; we call the vector $d$ the *update* of transition $s_1 \xrightarrow{d} s_2$. A *path* $\pi$ of $\mathcal{V}$ is a finite sequence $s_0 \xrightarrow{d_1} s_1 \xrightarrow{d_2} \cdots s_k$ with $s_i \xrightarrow{d_{i+1}} s_{i+1} \in \mathit{Trns}(\mathcal{V})$ for all $0 \leq i < k$. We define the *length* of $\pi$ by $\mathit{length}(\pi) = k$ and the *value* of $\pi$ by $\mathit{val}(\pi) = \sum_{i \in [1,k]} d_i$. Let $\mathtt{instance}(\pi, t)$ be the number of times $\pi$ contains the transition $t$, i.e., the number of indices $i$ such that $t = s_i \xrightarrow{d_i} s_{i+1}$. We remark that $\mathit{length}(\pi) = \sum_{t \in \mathit{Trns}(\mathcal{V})} \mathtt{instance}(\pi, t)$ for every path $\pi$ of $\mathcal{V}$. Given a finite path $\pi_1$ and a path $\pi_2$ such that the last state of $\pi_1$ equals the first state of $\pi_2$, we write $\pi = \pi_1\pi_2$ for the path obtained by joining the last state of $\pi_1$ with the first state of $\pi_2$; we call $\pi$ the *concatenation* of $\pi_1$ and $\pi_2$, and $\pi_1\pi_2$ a *decomposition* of $\pi$. We say $\pi'$ is a *sub-path* of $\pi$, if there is a decomposition $\pi = \pi_1\pi'\pi_2$ for some $\pi_1, \pi_2$. A *cycle* is a path that has the same start- and end-state. A *multi-cycle* is a finite set of cycles. The value $\mathit{val}(M)$ of a multi-cycle $M$ is the sum of the values of its cycles. $\mathcal{V}$ is *connected*, if for all $s, s' \in \mathit{St}(\mathcal{V})$ there is a path from $s$ to $s'$. VASS $\mathcal{V}'$ is a *sub-VASS* of $\mathcal{V}$, if $\mathit{St}(\mathcal{V}') \subseteq \mathit{St}(\mathcal{V})$ and $\mathit{Trns}(\mathcal{V}') \subseteq \mathit{Trns}(\mathcal{V})$. Sub-VASSs $\mathcal{V}_1$ and $\mathcal{V}_2$ are *disjoint*, if $\mathit{St}(\mathcal{V}_1) \cap \mathit{St}(\mathcal{V}_2) = \emptyset$. A *strongly-connected component (SCC)* of a VASS $\mathcal{V}$ is a maximal sub-VASS $S$ of $\mathcal{V}$ such that $S$ is connected and $\mathit{Trns}(S) \neq \emptyset$.

Let $\mathcal{V}$ be a VASS. The set of *valuations* $\mathit{Val}(\mathcal{V}) = \mathbb{N}^{\mathit{Var}}$ consists of $\mathit{Var}$-vectors over the natural numbers (we assume $\mathbb{N}$ includes 0). The set of *configurations* $\mathit{Cfg}(\mathcal{V}) = \mathit{St}(\mathcal{V}) \times \mathit{Val}(\mathcal{V})$ consists of pairs of states and valuations. A *step* is a triple $((s_1, \nu_1), d, (s_2, \nu_2)) \in \mathit{Cfg}(\mathcal{V}) \times \mathbb{Z}^{\mathit{dim}(\mathcal{V})} \times \mathit{Cfg}(\mathcal{V})$ such that $\nu_2 = \nu_1 + d$ and $s_1 \xrightarrow{d} s_2 \in \mathit{Trns}(\mathcal{V})$. We write $(s_1, \nu_1) \xrightarrow{d} (s_2, \nu_2)$ to denote a step $((s_1, \nu_1), d, (s_2, \nu_2))$ of $\mathcal{V}$. A *trace* of $\mathcal{V}$ is a finite sequence $\zeta = (s_0, \nu_0) \xrightarrow{d_1} (s_1, \nu_1) \xrightarrow{d_2} \cdots (s_k, \nu_k)$ of steps. We lift the notions of length and instances from paths to traces in the obvious way: we consider the path $\pi = s_0 \xrightarrow{d_1} s_1 \xrightarrow{d_2} \cdots s_k$ that consists of the transitions used by $\zeta$, and set $\mathit{length}(\zeta) := \mathit{length}(\pi)$ and $\mathtt{instance}(\zeta, t) = \mathtt{instance}(\pi, t)$, for all $t \in \mathit{Trns}(\mathcal{V})$. We denote by $\mathtt{init}(\zeta) = \|\nu_0\|$ the maximum absolute value of the starting valuation $\nu_0$ of $\zeta$. We say that $\zeta$ *reaches* a valuation $\nu$, if $\nu = \nu_k$. The *complexity* of $\mathcal{V}$ is the function $comp_{\mathcal{V}}(N) = \sup_{\text{trace } \zeta \text{ of } \mathcal{V}, \texttt{init}(\zeta) \leq N} length(\zeta)$, which returns for every $N \geq 0$ the supremum over the lengths of the traces $\zeta$ with $\texttt{init}(\zeta) \leq N$. The *variable bound* of a variable $x \in Var$ is the function $\texttt{vbound}_x(N) = \sup_{\text{trace } \zeta \text{ of } \mathcal{V}, \texttt{init}(\zeta) \leq N, \zeta \text{ reaches valuation } \nu} \nu(x)$, which returns for every $N \geq 0$ the supremum over the the values of $x$ reachable by traces $\zeta$ with $\texttt{init}(\zeta) \leq N$. The *transition bound* of a transition $t \in Trns(\mathcal{V})$ is the function $\texttt{tbound}_t(N) = \sup_{\text{trace } \zeta \text{ of } \mathcal{V}, \texttt{init}(\zeta) \leq N} \texttt{instance}(\zeta, t)$, which returns for every $N \geq 0$ the supremum over the number of instances of $t$ in traces $\zeta$ with $\texttt{init}(\zeta) \leq N$.

*Rooted Tree.* A *rooted tree* is a connected undirected acyclic graph in which one node has been designated as the root. We will usually denote the root by $\iota$. We note that for every node $\eta$ in a rooted tree there is a unique path of $\eta$ to the root. The *parent* of a node $\eta \neq \iota$ is the node connected to $\eta$ on the path to the root. Node $\eta$ is a *child* of a node $\eta'$, if $\eta'$ is the parent of $\eta$. $\eta'$ is a *descendent* of $\eta$, if $\eta$ lies on the path from $\eta'$ to the root; $\eta'$ is a *strict* descendent, if furthermore $\eta \neq \eta'$. $\eta$ is an *ancestor* of $\eta'$, if $\eta'$ a descendent of $\eta$; $\eta$ is a *strict* ancestor, if furthermore $\eta \neq \eta'$. The *distance* of a node $\eta$ to the root, is the number of nodes $\neq \eta$ on the path from $\eta$ to the root. We denote by $\texttt{layer}(l)$ the set of all nodes with the same distance $l$ to the root; we remark that $\texttt{layer}(0) = \{\iota\}$.

All proofs are presented in the extended version [21] for space reasons.

## 3 A Dichotomy Result

We will make use of the following matrices associated to a VASS throughout the paper: Let $\mathcal{V}$ be a VASS. We define the *update matrix* $D \in \mathbb{Z}^{Var \times Trns(\mathcal{V})}$ by setting $D(t) = d$ for all transitions $t = (s, d, s') \in Trns(\mathcal{V})$. We define the *flow matrix* $F \in \mathbb{Z}^{St(\mathcal{V}) \times Trns(\mathcal{V})}$ by setting $F(s, t) = -1$, $F(s', t) = 1$ for transitions $t = (s, d, s')$ with $s' \neq s$, and $F(s, t) = F(s', t) = 0$ for transitions $t = (s, d, s')$ with $s' = s$; in both cases we further set $F(s'', t) = 0$ for all states $s''$ with $s'' \neq s$ and $s'' \neq s'$. We note that every column $t$ of $F$ either contains exactly one $-1$ and 1 entry (in case the source and target of transition $t$ are different) or only 0 entries (in case the source and target of transition $t$ are the same).

*Example 1.* We state the update and flow matrix for $\mathcal{V}_{run}$ from Section 1:

$$D = \begin{pmatrix} -1 & 1 & -1 & 1 & 0 & 0 & 0 & 0 & -1 & 0 \\ 1 & -1 & 1 & -1 & 0 & 0 & 0 & 0 & 0 & 0 \\ -1 & 1 & 1 & -1 & -1 & -1 & -1 & -1 & 0 & 0 \end{pmatrix}, F = \begin{pmatrix} 0 & 0 & 0 & 0 & 1 & -1 & 0 & 0 & -1 & 0 \\ 0 & 0 & 0 & 0 & -1 & 1 & 0 & 0 & 0 & 1 \\ 0 & 0 & 0 & 0 & 0 & 0 & 1 & -1 & 1 & 0 \\ 0 & 0 & 0 & 0 & 0 & 0 & -1 & 1 & 0 & -1 \end{pmatrix},$$

with column order $s_1 \to s_1$, $s_2 \to s_2$, $s_3 \to s_3$, $s_4 \to s_4$, $s_2 \to s_1$, $s_1 \to s_2$, $s_4 \to s_3$, $s_3 \to s_4$, $s_1 \to s_3$, $s_4 \to s_2$ (from left to right) and row order $x, y, z$ for $D$ resp. $s_1, s_2, s_3, s_4$ for $F$ (from top to bottom).

We now consider the constraint systems ($P$) and ($Q$), stated below, which have maximization objectives. The constraint systems will be used by our main algorithm in Section 4. We observe that both constraint systems are always satisfiable (set all coefficients to zero) and that the solutions of both constraint systems are closed under addition. Hence, the number of inequalities for which the maximization objective is satisfied is unique for optimal solutions of both constraint systems. The maximization objectives can be implemented by suitable linear objective functions. Hence, both constraint systems can be solved in PTIME over the integers, because we can use linear programming over the rationales and then scale rational solutions to the integers by multiplying with the least common multiple of the denominators.

| constraint system $(P)$: | constraint system $(Q)$: |
|---|---|
| there exists $\mu \in \mathbb{Z}^{Trns(\mathcal{V})}$ with | there exist $r \in \mathbb{Z}^{Var}, z \in \mathbb{Z}^{St(\mathcal{V})}$ with |
| $D\mu \geq 0$ <br> $\mu \geq 0$ <br> $F\mu = 0$ | $r \geq 0$ <br> $z \geq 0$ <br> $D^T r + F^T z \leq 0$ |
| Maximization Objective: <br> Maximize the number of inequalities with $(D\mu)(x) > 0$ and $\mu(t) > 0$ | Maximization Objective: <br> Maximize the number of inequalities with $r(x) > 0$ and $(D^T r + F^T z)(t) < 0$ |

The solutions of $(P)$ and $(Q)$ are characterized by the following two lemmata:

**Lemma 2 (Cited from [14]).** *$\mu \in \mathbb{Z}^{Trns(\mathcal{V})}$ is a solution to constraint system $(P)$ iff there exists a multi-cycle $M$ with $val(M) \geq 0$ and $\mu(t)$ instances of transition $t$ for every $t \in Trns(\mathcal{V})$.*

**Lemma 3 (Cited from [5][1]).** *Let $r, z$ be a solution to constraint system $(Q)$. Let $rank(r, z) : Cfg(\mathcal{V}) \to \mathbb{N}$ be the function defined by $rank(r, z)(s, \nu) = r^T \nu + z(s)$. Then, $rank(r, z)$ is a* quasi-ranking function *for $\mathcal{V}$, i.e., we have*

1. *for all $(s, \nu) \in Cfg(\mathcal{V})$ that $rank(r, z)(s, \nu) \geq 0$;*
2. *for all transitions $t = s_1 \xrightarrow{d} s_2 \in Trns(\mathcal{V})$ and valuations $\nu_1, \nu_2 \in Val(\mathcal{V})$ with $\nu_2 = \nu_1 + d$ that $rank(r, z)(s_1, \nu_1) \geq rank(r, z)(s_2, \nu_2)$; moreover, the inequality is strict for every $t$ with $(D^T r + F^T z)(t) < 0$.*

We now state a dichotomy between optimal solutions to constraint systems $(P)$ and $(Q)$, which is obtained by an application of Farkas' Lemma. This dichotomy is the main reason why we are able to compute the precise asymptotic complexity of VASSs with polynomial bounds.

[1] There is no explicit lemma with this statement in [5], however the lemma is implicit in the exposition of Section 4 in [5]. We further note that [5] does not include the constraint $z \geq 0$. However, this difference is minor and was added in order to ensure that ranking functions always return non-negative values, which is more standard than the choice of [5]. A proof of the lemma can be found in the extended version [21].

**Lemma 4.** *Let $r$ and $z$ be an optimal solution to constraint system $(Q)$ and let $\mu$ be an optimal solution to constraint system $(P)$. Then, for all variables $x \in Var$ we either have $r(x) > 0$ or $(D\mu)(x) \geq 1$, and for all transitions $t \in Trns(\mathcal{V})$ we either have $(D^T r + F^T z)(t) < 0$ or $\mu(t) \geq 1$.*

*Example 5.* Our main algorithm, Algorithm 1 presented in Section 4, will directly use constraint systems $(P)$ and $(Q)$ in its first loop iteration, and adjusted versions in later loop iterations. Here, we illustrate the first loop iteration. We consider the running example $\mathcal{V}_{run}$, whose update and flow matrices we have stated in Example 1. An optimal solution to constraint systems $(P)$ and $(Q)$ is given by $\mu = (1441111100)^T$ and $r = (220)^T$, $z = (0011)^T$. The quasi-ranking function $rank(r, z)$ immediately establishes that $\texttt{tbound}_t(N) \in O(N)$ for $t = s_1 \rightarrow s_3$ and $t = s_4 \rightarrow s_2$, because 1) $rank(r, z)$ decreases for these two transitions and does not increase for other transitions (by Lemma 3), and because 2) the initial value of $rank(r, z)$ is bounded by $O(N)$, i.e., we have $rank(r, z)(s, \nu) \in O(N)$ for every state $s \in St(\mathcal{V}_{run})$ and every valuation $\nu$ with $\|\nu\| \leq N$. By a similar argument we get $\texttt{vbound}_x(N) \in O(N)$ and $\texttt{vbound}_y(N) \in O(N)$. The exact reasoning for deriving upper bounds is given in Section 5. From $\mu$ we can, by Lemma 2, obtain the cycles $C_1 = s_1 \rightarrow s_2 \rightarrow s_2 \rightarrow s_2 \rightarrow s_2 \rightarrow s_2 \rightarrow s_1 \rightarrow s_1$ and $C_2 = s_3 \rightarrow s_4 \rightarrow s_4 \rightarrow s_4 \rightarrow s_4 \rightarrow s_4 \rightarrow s_4 \rightarrow s_4$ with $\nu(C_1) + \nu(C_2) \geq (001)^T$ (*). We will later show that the cycles $C_1$ and $C_2$ give rise to a family of traces that establish $\texttt{tbound}_t(N) \in \Omega(N^2)$ for all transitions $t \in Trns(\mathcal{V}_{run})$ with $t \neq s_1 \rightarrow s_3$ and $t \neq s_4 \rightarrow s_2$. Here we give an intuition on the construction: We consider a cycle $C$ of $\mathcal{V}_{run}$ that visits all states at least once. By (*), the updates along the cycles $C_1$ and $C_2$ cancel each other out. However, the two cycles are not connected. Hence, we execute the cycle $C_1$ some $\Omega(N)$ times, then (a part of) the cycle $C$, then execute $C_2$ as often as $C_1$, and finally the remaining part of $C$; this we repeat $\Omega(N)$ times. This construction also establishes the bound $\texttt{vbound}_z(N) \in \Omega(N^2)$ because, by (*), we increase $z$ with every joint execution of $C_1$ and $C_2$. The precise lower bound construction is given in Section 6.

## 4 Main Algorithm

Our main algorithm – Algorithm 1 – computes the complexity as well as variable and transition bounds of an input VASS $\mathcal{V}$, either detecting that $\mathcal{V}$ has at least exponential complexity or reporting precise asymptotic bounds for the transitions and variables of $\mathcal{V}$ (up to a constant factor): Algorithm 1 will compute values $\texttt{vExp}(x) \in \mathbb{N}$ such that $\texttt{vbound}_N(x) \in \Theta(N^{\texttt{vExp}(x)})$ for every $x \in Var$ and values $\texttt{tExp}(t) \in \mathbb{N}$ such that $\texttt{tbound}_N(t) \in \Theta(N^{\texttt{tExp}(t)})$ for every $t \in Trns(\mathcal{V})$.

*Data Structures.* The algorithm maintains a rooted tree $T$. Every node $\eta$ of $T$ will always be labelled by a sub-VASSs $\texttt{VASS}(\eta)$ of $\mathcal{V}$. The nodes in the same layer of $T$ will always be labelled by disjoint sub-VASS of $\mathcal{V}$. The main loop of Algorithm 1 will extend $T$ by one layer per loop iteration. The variable $l$ always contains the next layer that is going to be added to $T$. For computing variable and transition bounds, Algorithm 1 maintains the functions $\texttt{vExp} : Var \rightarrow \mathbb{N} \cup \{\infty\}$ and $\texttt{tExp} : Trns(\mathcal{V}) \rightarrow \mathbb{N} \cup \{\infty\}$.

*Initialization.* We assume $D$ to be the update matrix and $F$ to be the flow matrix associated to $\mathcal{V}$ as discussed in Section 3. At initialization, $T$ consists of the root node $\iota$ and we set $\texttt{VASS}(\iota) = \mathcal{V}$, i.e., the root is labelled by the input $\mathcal{V}$. We initialize $l = 1$ as Algorithm 1 is going to add layer 1 to $T$ in the first loop iteration. We initialize $\texttt{vExp}(x) = \infty$ for all variables $x \in \mathit{Var}$ and $\texttt{tExp}(t) = \infty$ for all transitions $t \in \mathit{Trns}(\mathcal{V})$.

*The constraint systems solved during each loop iteration.* In loop iteration $l$, Algorithm 1 will set $\texttt{tExp}(t) := l$ for some transitions $t$ and $\texttt{vExp}(x) := l$ for some variables $x$. In order to determine those transitions and variables, Algorithm 1 instantiates constraint systems $(P)$ and $(Q)$ from Section 3 over the set of transitions $U = \bigcup_{\eta \in \texttt{layer}(l-1)} \mathit{Trns}(\texttt{VASS}(\eta))$, which contains all transitions associated to nodes in layer $l-1$ of $T$. However, instead of a direct instantiation using $D|_U$ and $F|_U$ (i.e., the restriction of $D$ and $F$ to the transitions $U$), we need to work with an extended set of variables and an extended update matrix. We set $\mathit{Var}_{ext} := \{(x, \eta) \mid \eta \in \texttt{layer}(l - \texttt{vExp}(x))\}$, where we set $n - \infty = 0$ for all $n \in \mathbb{N}$. This means that we use a different copy of variable $x$ for every node $\eta$ in layer $l - \texttt{vExp}(x)$. We note that for a variable $x$ with $\texttt{vExp}(x) = \infty$ there is only a single copy of $x$ in $\mathit{Var}_{ext}$ because $\iota \in \texttt{layer}(0)$ is the only node in layer 0. We define the extended update matrix $D_{ext} \in \mathbb{Z}^{\mathit{Var}_{ext} \times U}$ by setting

$$D_{ext}((x, \eta), t) := \begin{cases} D(x, t), \text{ if } t \in \mathit{Trns}(\texttt{VASS}(\eta)), \\ \quad 0, \qquad\qquad \text{otherwise.} \end{cases}$$

Constraint systems $(I)$ and $(II)$ stated in Figure 2 can be recognized as instantiation of constraint systems $(P)$ and $(Q)$ with matrices $D_{ext}$ and $F|_U$ and variables $\mathit{Var}_{ext}$, and hence the dichotomy stated in Lemma 4 holds.

We comment on the choice of $\mathit{Var}_{ext}$: Setting $\mathit{Var}_{ext} = \{(x, \eta) \mid \eta \in \texttt{layer}(i)\}$ for any $i \leq l - \texttt{vExp}(x)$ would result in correct upper bounds (while $i > l - \texttt{vExp}(x)$ would not). However, choosing $i < l - \texttt{vExp}(x)$ does in general result in sub-optimal bounds because fewer variables make constraint system $(I)$ easier and constraint system $(II)$ harder to satisfy (in terms of their maximization objectives). In fact, $i = l - \texttt{vExp}(x)$ is the optimal choice, because this choice allows us to prove corresponding lower bounds in Section 6. We will further comment on key properties of constraint systems $(I)$ and $(II)$ in Sections 5 and 6, when we outline the proofs of the upper resp. lower bound.

We note that Algorithm 1 does not use the optimal solution $\mu$ to constraint system $(I)$ for the computation of the $\texttt{vExp}(x)$ and $\texttt{tExp}(t)$, and hence the computation of the optimal solution $\mu$ could be removed from the algorithm. The solution $\mu$ is however needed for the extraction of lower bounds in Sections 6 and 8, and this is the reason why it is stated here. The extraction of lower bounds is not explicitly added to the algorithm in order to not clutter the presentation.

*Discovering transition bounds.* After an optimal solution $r$, $z$ to constraint system $(II)$ has been found, Algorithm 1 collects all transitions $t$ with $(D_{ext}^T r + F|_U^T z)(t) < 0$ in the set $R$ (note that the optimization criterion in constraint system $(II)$ tries to find as many such $t$ as possible). Algorithm 1 then sets $\texttt{tExp}(t) := l$ for all $t \in R$. The transitions in $R$ will not be part of layer $l$ of $T$.

**Input:** a connected VASS $\mathcal{V}$ with update matrix $D$ and flow matrix $F$
$T :=$ single root node $\iota$ with $\texttt{VASS}(\iota) = \mathcal{V}$;
$l := 1$;
$\texttt{vExp}(x) := \infty$ for all variables $x \in \mathit{Var}$;
$\texttt{tExp}(t) := \infty$ for all transitions $t \in \mathit{Trns}(\mathcal{V})$;
**repeat**
  let $U := \bigcup_{\eta \in \texttt{layer}(l-1)} \mathit{Trns}(\texttt{VASS}(\eta))$;
  let $\mathit{Var}_{ext} := \{(x, \eta) \mid \eta \in \texttt{layer}(l - \texttt{vExp}(x))\}$, where $n - \infty = 0$ for $n \in \mathbb{N}$;
  let $D_{ext} \in \mathbb{Z}^{\mathit{Var}_{ext} \times U}$ be the matrix defined by
  $$D_{ext}((x,\eta),t) = \begin{cases} D(x,t), & \text{if } t \in \mathit{Trns}(\texttt{VASS}(\eta)) \\ 0, & \text{otherwise} \end{cases};$$
  find optimal solutions $\mu$ and $r$, $z$ to constraint systems $(I)$ and $(II)$;
  let $R := \{t \in U \mid (D_{ext}^T r + F|_U^T z)(t) < 0\}$;
  set $\texttt{tExp}(t) := l$ for all $t \in R$;
  **foreach** $\eta \in \texttt{layer}(l-1)$ **do**
    let $\mathcal{V}' := \texttt{VASS}(\eta)$ be the VASS associated to $\eta$;
    decompose $(\mathit{St}(\mathcal{V}'), \mathit{Trns}(\mathcal{V}') \setminus R)$ into SCCs;
    **foreach** *SCC* $S$ *of* $(\mathit{St}(\mathcal{V}'), \mathit{Trns}(\mathcal{V}') \setminus R)$ **do**
      create a child $\eta'$ of $\eta$ with $\texttt{VASS}(\eta') = S$;
  **foreach** $x \in \mathit{Var}$ *with* $\texttt{vExp}(x) = \infty$ **do**
    **if** $r(x, \iota) > 0$ **then** set $\texttt{vExp}(x) := l$ ;
  **if** *there are no* $x \in \mathit{Var}$, $t \in \mathit{Trns}(\mathcal{V})$ *with* $l < \texttt{vExp}(x) + \texttt{tExp}(t) < \infty$ **then**
    **return** "$\mathcal{V}$ has at least exponential complexity"
  $l := l + 1$;
**until** $\texttt{vExp}(x) \neq \infty$ *and* $\texttt{tExp}(t) \neq \infty$ *for all* $x \in \mathit{Var}$ *and* $t \in \mathit{Trns}(\mathcal{V})$;

**Algorithm 1:** Computes transition and variable bounds for a VASS $\mathcal{V}$

| constraint system $(I)$: | constraint system $(II)$: |
|---|---|
| there exists $\mu \in \mathbb{Z}^U$ with | there exist $r \in \mathbb{Z}^{\mathit{Var}_{ext}}, z \in \mathbb{Z}^{\mathit{St}(\mathcal{V})}$ with |
| $D_{ext}\mu \geq 0$ <br> $\mu \geq 0$ <br> $F\|_U \mu = 0$ | $r \geq 0$ <br> $z \geq 0$ <br> $D_{ext}^T r + F\|_U^T z \leq 0$ |
| Maximization Objective: Maximize the number of inequalities with $(D_{ext}\mu)(x) > 0$ and $\mu(t) > 0$ | Maximization Objective: Maximize the number of inequalities with $r(x,\eta) > 0$ and $(D_{ext}^T r + F\|_U^T z)(t) < 0$ |

**Fig. 2.** Constraint Systems $(I)$ and $(II)$ used by Algorithm 1

*Construction of the next layer in $T$.* For each node $\eta$ in layer $l-1$, Algorithm 1 will create children by removing the transitions in $R$. This is done as follows: Given a node $\eta$ in layer $l-1$, Algorithm 1 considers the VASS $\mathcal{V}' = \texttt{VASS}(\eta)$ associated to $\eta$. Then, $(\mathit{St}(\mathcal{V}'), \mathit{Trns}(\mathcal{V}') \setminus R)$ is decomposed into its SCCs. Finally, for each SCC $S$ of $(St(\mathcal{V}'), \mathit{Trns}(\mathcal{V}') \setminus R)$ a child $\eta'$ of $\eta$ is created with $\mathtt{VASS}(\eta') = S$. Clearly, the new nodes in layer $l$ are labelled by disjoint sub-VASS of $\mathcal{V}$.

*The transitions of the next layer.* The following lemma states that the new layer $l$ of $T$ contains all transitions of layer $l-1$ except for the transitions $R$; the lemma is due to the fact that every transition in $U \setminus R$ belongs to a cycle and hence to some SCC that is part of the new layer $l$.

**Lemma 6.** *We consider the new layer constructed during loop iteration $l$ of Algorithm 1: we have $U \setminus R = \bigcup_{\eta \in \mathtt{layer}(l)} \mathit{Trns}(\mathtt{VASS}(\eta))$.*

*Discovering variable bounds.* For each $x \in \mathit{Var}$ with $\mathtt{vExp}(x) = \infty$, Algorithm 1 checks whether $r(x, \iota) > 0$ (we point out that the optimization criterion in constraint systems $(II)$ tries to find as many such $x$ with $r(x, \iota) > 0$ as possible). Algorithm 1 then sets $\mathtt{vExp}(x) := l$ for all those variables.

*The check for exponential complexity.* In each loop iteration, Algorithm 1 checks whether there are $x \in \mathit{Var}$, $t \in \mathit{Trns}(\mathcal{V})$ with $l < \mathtt{vExp}(x) + \mathtt{tExp}(t) < \infty$. If this is not the case, then we can conclude that $\mathcal{V}$ is at least exponential (see Theorem 9 below). If the check fails, Algorithm 1 increments $l$ and continues with the construction of the next layer in the next loop iteration.

*Termination criterion.* The algorithm proceeds until either exponential complexity has been detected or until $\mathtt{vExp}(x) \neq \infty$ and $\mathtt{tExp}(t) \neq \infty$ for all $x \in \mathit{Var}$ and $t \in \mathit{Trns}(\mathcal{V})$ (i.e., bounds have been computed for all variables and transitions).

*Invariants.* We now state some simple invariants maintained by Algorithm 1, which are easy to verify:

- For every node $\eta$ that is a descendent of some node $\eta'$ we have that $\mathtt{VASS}(\eta)$ is a sub-VASS of $\mathtt{VASS}(\eta')$.
- The value of $\mathtt{vExp}$ and $\mathtt{tExp}$ is changed at most once for each input; when the value is changed, it is changed from $\infty$ to some value $\neq \infty$.
- For every transition $t \in \mathit{Trns}(\mathcal{V})$ and layer $l$ of $T$, we have that either $\mathtt{tExp}(t) \leq l$ or there is a node $\eta \in \mathtt{layer}(l)$ such that $t \in \mathit{Trns}(\mathtt{VASS}(\eta))$.
- We have $\mathtt{tExp}(t) = l$ for $t \in \mathit{Trns}(\mathcal{V})$ if and only if there is a $\eta \in \mathtt{layer}(l-1)$ with $t \in \mathit{Trns}(\mathtt{VASS}(\eta))$ and there is no $\eta \in \mathtt{layer}(l)$ with $t \in \mathit{Trns}(\mathtt{VASS}(\eta))$.

*Example 7.* We sketch the execution of Algorithm 1 on $\mathcal{V}_{run}$. In iteration $l = 1$, we have $\mathit{Var}_{ext} = \{(x, \iota), (y, \iota), (z, \iota)\}$, and thus matrix $D_{ext}$ is identical to the matrix $D$. Hence, constraint systems $(I)$ and $(II)$ are identical to constraint systems $(P)$ and $(Q)$, whose optimal solutions $\mu = (1441111100)^T$ and $r = (220)^T$, $z = (0011)^T$ we have discussed in Example 5. Algorithm 1 then sets $\mathtt{tExp}(s_1 \to s_3) = 1$ and $\mathtt{tExp}(s_4 \to s_2) = 1$, creates two children $\eta_A$ and $\eta_B$ of $\iota$ labeled by $\mathcal{V}_A = (\{s_1, s_2\}, \{s_1 \to s_1, s_1 \to s_2, s_2 \to s_2, s_2 \to s_1\})$ and $\mathcal{V}_B = (\{s_3, s_4\}, \{s_3 \to s_3, s_3 \to s_4, s_4 \to s_4, s_4 \to s_3\})$, and sets $\mathtt{vExp}(x) = 1$ and $\mathtt{vExp}(y) = 1$. In iteration $l = 2$, we have $\mathit{Var}_{ext} = \{(x, \eta_A), (y, \eta_A), (x, \eta_B), (y, \eta_B), (z, \iota)\}$ and the matrix $D_{ext}$ stated in Figure 3. Algorithm 1 obtains $\mu = (11110000)^T$ and $r = (12211)^T$, $z = (0000)^T$ as optimal solutions to $(I)$ and $(II)$. Algorithm 1 then

$$D_{ext} = \begin{pmatrix} -1 & 1 & 0 & 0 & 0 & 0 & 0 & 0 \\ 1 & -1 & 0 & 0 & 0 & 0 & 0 & 0 \\ 0 & 0 & -1 & 1 & 0 & 0 & 0 & 0 \\ 0 & 0 & 1 & -1 & 0 & 0 & 0 & 0 \\ -1 & 1 & 1 & -1 & -1 & -1 & -1 & -1 \end{pmatrix}$$

with column order $s_1 \to s_1$, $s_2 \to s_2$, $s_3 \to s_3$, $s_4 \to s_4$, $s_2 \to s_1$, $s_1 \to s_2$, $s_4 \to s_3$, $s_3 \to s_4$ (from left to right) and row order $(x, \eta_A), (y, \eta_A), (x, \eta_B), (y, \eta_B), (z, \iota)$ (from top to bottom)

$$D_{ext} = \begin{pmatrix} -1 & 0 & 0 & 0 \\ 1 & 0 & 0 & 0 \\ 0 & 1 & 0 & 0 \\ 0 & -1 & 0 & 0 \\ 0 & 0 & -1 & 0 \\ 0 & 0 & 1 & 0 \\ 0 & 0 & 0 & 1 \\ 0 & 0 & 0 & -1 \\ -1 & 1 & 0 & 0 \\ 0 & 0 & 1 & -1 \end{pmatrix}$$

with column order $s_1 \to s_1$, $s_2 \to s_2$, $s_3 \to s_3$, $s_4 \to s_4$, (from left to right) and row order $(x, \eta_1), (y, \eta_1), (x, \eta_2), (y, \eta_2), (x, \eta_3), (y, \eta_3), (x, \eta_4), (y, \eta_4), (z, \eta_A), (z, \eta_B)$ (from top to bottom)

**Fig. 3.** The extended update matrices during iteration $l = 2$ (left) and $l = 3$ (right) of Algorithm 1 on the running example $\mathcal{V}_{run}$ from Section 1.

sets $\texttt{tExp}(s_1 \to s_2) = \texttt{tExp}(s_2 \to s_1) = \texttt{tExp}(s_3 \to s_4) = \texttt{tExp}(s_4 \to s_3) = 2$, creates the children $\eta_1, \eta_2$ resp. $\eta_3, \eta_4$ of $\eta_A$ resp. $\eta_B$ with $\eta_i$ labelled by $\mathcal{V}_i = (\{s_i\}, \{s_i \to s_i\})$, and sets $\texttt{vExp}(z) = 2$. In iteration $l = 3$, we have $Var_{ext} = \{(x, \eta_1), (y, \eta_1), (x, \eta_2), (y, \eta_2), (x, \eta_3), (y, \eta_3), (x, \eta_4), (y, \eta_4), (z, \eta_A), (z, \eta_B)\}$ and the matrix $D_{ext}$ stated in Figure 3. Algorithm 1 obtains $\mu = (0000)^T$ and $r = (1113311111)^T$, $z = (0000)^T$ as optimal solutions to $(I)$ and $(II)$. Algorithm 1 then sets $\texttt{tExp}(s_i \to s_i) = 3$, for all $i$, and terminates.

We now state the main properties of Algorithm 1:

**Lemma 8.** *Algorithm 1 always terminates.*

**Theorem 9.** *If Algorithm 1 returns "$\mathcal{V}$ has at least exponential complexity", then $comp_{\mathcal{V}}(N) \in 2^{\Omega(N)}$, and we have $\texttt{tbound}_t(N) \in 2^{\Omega(N)}$ for all $t \in Trns(\mathcal{V})$ with $\texttt{tExp}(t) = \infty$ and $\texttt{vbound}_t(N) \in 2^{\Omega(N)}$ for all $x \in Var$ with $\texttt{vExp}(x) = \infty$.*

The proof of Theorem 9 is stated in Section 8. We now assume that Algorithm 1 does not return "$\mathcal{V}$ has at least exponential complexity". Then, Algorithm 1 must terminate with $\texttt{tExp}(t) \neq \infty$ and $\texttt{vExp}(x) \neq \infty$ for all $t \in Trns(\mathcal{V})$ and $x \in Var$. The following result states that `tExp` and `vExp` contain the precise exponents of the asymptotic transition and variable bounds of $\mathcal{V}$:

**Theorem 10.** *$\texttt{vbound}_N(x) \in \Theta(N^{\texttt{vExp}(x)})$ for all $x \in Var$ and $\texttt{tbound}_N(t) \in \Theta(N^{\texttt{tExp}(t)})$ for all $t \in Trns(\mathcal{V})$.*

The upper bounds of Theorem 10 will be proved in Section 5 (Theorem 16) and the lower bounds in Section 6 (Corollary 20).

We will prove in Section 7 that the exponents of the variable and transition bounds are bounded exponentially in the dimension of $\mathcal{V}$:

**Theorem 11.** *We have $\texttt{vExp}(x) \leq 2^{|Var|}$ for all $x \in Var$ and $\texttt{tExp}(t) \leq 2^{|Var|}$ for all $t \in Trns(\mathcal{V})$.*

Finally, we obtain the following corollary from Theorems 10 and 11:

**Corollary 12.** *Let $\mathcal{V}$ be a connected VASS. Then, either $comp_{\mathcal{V}}(N) \in 2^{\Omega(N)}$ or $comp_{\mathcal{V}}(N) \in \Theta(N^i)$ for some computable $1 \leq i \leq 2^{|Var|}$.*

### 4.1 Complexity of Algorithm 1

In the remainder of this section we will establish the following result:

**Theorem 13.** *Algorithm 1 (with the below stated optimization) can be implemented in polynomial time with regard to the size of the input VASS $\mathcal{V}$.*

We will argue that A) every loop iteration of Algorithm 1 only takes polynomial time, and B) that polynomially many loop iterations are sufficient (this only holds for the optimization of the algorithm discussed below).

Let $\mathcal{V}$ be a VASS, let $m = |\mathit{Trns}(\mathcal{V})|$ be the number of transitions of $\mathcal{V}$, and let $n = |\mathit{Var}|$ be the dimension of $\mathcal{V}$. We note that $|\texttt{layer}(l)| \leq m$ for every layer $l$ of $T$, because the VASSs of the nodes in the same layer are disjoint.

A) Clearly, removing the decreasing transitions and computing the strongly connected components can be done in polynomial time. It remains to argue about constraint systems $(I)$ and $(II)$. We observe that $|\mathit{Var}_{ext}| = |\{(x,\eta) \mid \eta \in \texttt{layer}(l - \texttt{vExp}(x))\}| \leq n \cdot m$ and $|U| \leq m$. Hence the size of constraint systems $(I)$ and $(II)$ is polynomial in the size of $\mathcal{V}$. Moreover, constraint systems $(I)$ and $(II)$ can be solved in PTIME as noted in Section 3.

B) We do not a-priori have a bound on the number of iterations of the main loop of Algorithm 1. (Theorem 11 implies that the number of iterations is at most exponential; however, we do not use this result here). We will shortly state an improvement of Algorithm 1 that ensures that polynomially many iterations are sufficient. The underlying insight is that certain layers of the tree do not need to be constructed explicitly. This insight is stated in the lemma below:

**Lemma 14.** *We consider the point in time when the execution of Algorithm 1 reaches line $l := l + 1$ during some loop iteration $l \geq 1$. Let $\mathit{RelevantLayers} = \{\texttt{tExp}(t) + \texttt{vExp}(x) \mid x \in \mathit{Var}, t \in \mathit{Trns}(\mathcal{V})\}$ and let $l' = \min\{l' \mid l' > l, l' \in \mathit{RelevantLayers}\}$. Then, $\texttt{vExp}(x) \neq i$ and $\texttt{tExp}(t) \neq i$ for all $x \in \mathit{Var}$, $t \in \mathit{Trns}(\mathcal{V})$ and $l < i < l'$.*

We now present the optimization that achieves polynomially many loop iterations. We replace the line $l := l+1$ by the two lines $\mathit{RelevantLayers} := \{\texttt{tExp}(t) + \texttt{vExp}(x) \mid x \in \mathit{Var}, t \in \mathit{Trns}(\mathcal{V})\}$ and $l := \min\{l' \mid l' > l, l' \in \mathit{RelevantLayers}\}$. The effect of these two lines is that Algorithm 1 directly skips to the next relevant layer. Lemma 14, stated above, justifies this optimization: First, no new variable or transition bound is discovered in the intermediate layers $l < i < l'$. Second, each intermediate layer $l < i < l'$ has the same number of nodes as layer $l$, which are labelled by the same sub-VASSs as the nodes in $l$ (otherwise there would be a transition with transition bound $l < i < l'$); hence, whenever needed, Algorithm 1 can construct a missing layer $l < i < l'$ on-the-fly from layer $l$.

We now analyze the number of loop iterations of the optimized algorithm. We recall that the value of each $\texttt{vExp}(x)$ and $\texttt{tExp}(t)$ is changed at most once from $\infty$ to some value $\neq \infty$. Hence, Algorithm 1 encounters at most $n \cdot m$ different values in the set $\mathit{RelevantLayers} = \{\texttt{tExp}(t) + \texttt{vExp}(x) \mid x \in \mathit{Var}, t \in \mathit{Trns}(\mathcal{V})\}$ during execution. Thus, the number of loop iterations is bounded by $n \cdot m$.

## 5 Proof of the Upper Bound Theorem

We begin by stating a proof principle for obtaining upper bounds.

**Proposition 15 (Bound Proof Principle).** *Let $\mathcal{V}$ be a VASS. Let $U \subseteq Trns(\mathcal{V})$ be a subset of the transitions of $\mathcal{V}$. Let $w : Cfg(\mathcal{V}) \to \mathbb{N}$ and $\texttt{inc}_t : \mathbb{N} \to \mathbb{N}$, for every $t \in Trns(\mathcal{V}) \setminus U$, be functions such that for every trace $\zeta = (s_0, \nu_0) \xrightarrow{d_1} (s_1, \nu_1) \xrightarrow{d_2} \cdots$ of $\mathcal{V}$ with $\texttt{init}(\zeta) \le N$ we have for every $i \ge 0$ that*

*1) $s_i \xrightarrow{d_i} s_{i+1} \in U$ implies $w(s_i, \nu_i) \ge w(s_{i+1}, \nu_{i+1})$, and*
*2) $s_i \xrightarrow{d_i} s_{i+1} \in Trns(\mathcal{V}) \setminus U$ implies $w(s_i, \nu_i) + \texttt{inc}_t(N) \ge w(s_{i+1}, \nu_{i+1})$.*

*We call such a function $w$ a* complexity witness *and the associated $\texttt{inc}_t$ functions the* increase certificates*.*

*Let $t \in U$ be a transition on which $w$* decreases*, i.e., we have $w(s_1, \nu_1) \ge w(s_2, \nu_2) - 1$ for every step $(s_1, \nu_1) \xrightarrow{d} (s_2, \nu_2)$ of $\mathcal{V}$ with $t = s_1 \xrightarrow{d} s_2$. Then,*

$$\texttt{tbound}_t(N) \le \max_{(s,\nu) \in Cfg(\mathcal{V}), \|\nu\| \le N} w(s, \nu) + \sum_{t' \in Trns(\mathcal{V}) \setminus U} \texttt{tbound}_{t'}(N) \cdot \texttt{inc}_{t'}(N).$$

*Further, let $x \in Var$ be a variable such that $\nu(x) \le w(s, \nu)$ for all $(s, \nu) \in Cfg(\mathcal{V})$. Then,*

$$\texttt{vbound}_x(N) \le \max_{(s,\nu) \in Cfg(\mathcal{V}), \|\nu\| \le N} w(s, \nu) + \sum_{t' \in Trns(\mathcal{V}) \setminus U} \texttt{tbound}_{t'}(N) \cdot \texttt{inc}_{t'}(N).$$

*Proof Outline of the Upper Bound Theorem.* Let $\mathcal{V}$ be a VASS for which Algorithm 1 does not report exponential complexity. We will prove by induction on loop iteration $l$ that $\texttt{vbound}_N(x) \in O(N^l)$ for every $x \in Var$ with $\texttt{vExp}(x) = l$ and that $\texttt{tbound}_N(t) \in O(N^l)$ for every $t \in Trns(\mathcal{V})$ with $\texttt{tExp}(t) = l$.

We now consider some loop iteration $l \ge 1$. Let $U = \bigcup_{\eta \in \texttt{layer}(l-1)} Trns(\texttt{VASS}(\eta))$ be the transitions, $Var_{ext}$ be the set of extended variables and $D_{ext} \in \mathbb{Z}^{Var_{ext} \times U}$ be the update matrix considered by Algorithm 1 during loop iteration $l$. Let $r, z$ be some optimal solution to constraint system $(II)$ computed by Algorithm 1 during loop iteration $l$. The main idea for the upper bound proof is to use the quasi-ranking function from Lemma 3 as witness function for the Bound Proof Principle. In order to apply Lemma 3 we need to consider the VASS associated to the matrices in constraint system $(II)$: Let $\mathcal{V}_{ext}$ be the VASS over variables $Var_{ext}$ associated to update matrix $D_{ext}$ and flow matrix $F|_U$. From Lemma 3 we get that $rank(r, z) : Cfg(\mathcal{V}_{ext}) \to \mathbb{N}$ is a quasi-ranking function for $\mathcal{V}_{ext}$. We now need to relate $\mathcal{V}$ to the extended VASS $\mathcal{V}_{ext}$ in order to be able to use this quasi-ranking function. We do so by extending valuations over $Var$ to valuations over $Var_{ext}$. For every state $s \in St(\mathcal{V})$ and valuation $\nu : Var \to \mathbb{N}$, we define the *extended valuation* $\texttt{ext}_s(\nu) : Var_{ext} \to \mathbb{N}$ by setting

$$\texttt{ext}_s(\nu)(x, \eta) = \begin{cases} \nu(x), & \text{if } s \in St(\texttt{VASS}(\eta)), \\ 0, & \text{otherwise.} \end{cases}$$

As a direct consequence from the definition of extended valuations, we have that $(s, \texttt{ext}_s(\nu)) \in \mathit{Cfg}(\mathcal{V}_{ext})$ for all $(s,\nu) \in \mathit{Cfg}(\mathcal{V})$, and that $(s_1, \texttt{ext}_{s_1}(\nu_1)) \xrightarrow{D_{ext}(t)} (s_2, \texttt{ext}_{s_2}(\nu_2))$ is a step of $\mathcal{V}_{ext}$ for every step $(s_1,\nu_1) \xrightarrow{d} (s_2,\nu_2)$ of $\mathcal{V}$ with $s_1 \xrightarrow{d} s_2 \in U$. We now define the witness function $w$ by setting

$$w(s,\nu) = \mathit{rank}(r,z)(s, \texttt{ext}_s(\nu)) \qquad \text{for all } (s,\nu) \in \mathit{Cfg}(\mathcal{V}).$$

We immediately get from Lemma 3 that $w$ maps configurations to the non-negative integers and that condition 1) of the Bound Proof Principle is satisfied. Indeed, we get from the first item of Lemma 3 that $w(s,\nu) \geq 0$ for all $(s,\nu) \in \mathit{Cfg}(\mathcal{V})$, and from the second item that $w(s_1,\nu_1) \geq w(s_2,\nu_2)$ for every step $(s_1,\nu_1) \xrightarrow{d} (s_2,\nu_2)$ of $\mathcal{V}$ with $t = s_1 \xrightarrow{d} s_2 \in U$; moreover, the inequality is strict if $(D_{ext}^T r + F|_U^T z)(t) < 0$, i.e., the witness function $w$ decreases for transitions $t$ with $\texttt{tExp}(t) = l$. It remains to establish condition 2) of the Bound Proof Principle. We will argue that we can find increase certificates $\texttt{inc}_t(N) \in O(N^{l-\texttt{tExp}(t)})$ for all $t \in \mathit{Trns}(\mathcal{V}) \setminus U$. We note that $\texttt{tExp}(t) < l$ for all $t \in \mathit{Trns}(\mathcal{V}) \setminus U$, and hence the induction assumption can be applied for such $t$. We can then derive the desired bounds from the Bound Proof Principle because of $\sum_{t \in \mathit{Trns}(\mathcal{V}) \setminus U} \texttt{tbound}_t(N) \cdot \texttt{inc}_t(N) = \sum_{t \in \mathit{Trns}(\mathcal{V}) \setminus U} O(N^{\texttt{tExp}(t)}) \cdot O(N^{l-\texttt{tExp}(t)}) = O(N^l)$.

**Theorem 16.** $\texttt{vbound}_N(x) \in O(N^{\texttt{vExp}(x)})$ *for all* $x \in \mathit{Var}$ *and* $\texttt{tbound}_N(t) \in O(N^{\texttt{tExp}(t)})$ *for all* $t \in \mathit{Trns}(\mathcal{V})$.

## 6 Proof of the Lower Bound Theorem

The following lemma will allow us to consider traces $\zeta_N$ with $\texttt{init}(\zeta_N) \in O(N)$ instead of $\texttt{init}(\zeta_N) \leq N$ when proving asymptotic lower bounds.

**Lemma 17.** *Let* $\mathcal{V}$ *be a VASS, let* $t \in \mathit{Trns}(\mathcal{V})$ *be a transition and let* $x \in \mathit{Var}$ *be a variable. If there are traces* $\zeta_N$ *with* $\texttt{init}(\zeta_N) \in O(N)$ *and* $\texttt{instance}(\zeta_N, t) \geq N^i$*, then* $\texttt{tbound}_N(t) \in \Omega(N^i)$*. If there are traces* $\zeta_N$ *with* $\texttt{init}(\zeta_N) \in O(N)$ *that reach a final valuation* $\nu$ *with* $\nu(x) \geq N^i$*, then* $\texttt{vbound}_N(x) \in \Omega(N^i)$*.*

The lower bound proof uses the notion of a *pre-path*, which relaxes the notion of a path: A pre-path $\sigma = t_1 \cdots t_k$ is a finite sequence of transitions $t_i = s_i \xrightarrow{d_i} s_i'$. Note that we do not require for subsequent transitions that the end state of one transition is the start state of the next transition, i.e., we do not require $s_i' = s_{i+1}$. We generalize notions from paths to pre-paths in the obvious way, e.g., we set $\mathit{val}(\sigma) = \sum_{i \in [1,k]} d_i$ and denote by $\texttt{instance}(\sigma, t)$, for $t \in \mathit{Trns}(\mathcal{V})$, the number of times $\sigma$ contains the transition $t$. We say the pre-path $\sigma$ *can be executed from valuation* $\nu$, if there are valuations $\nu_i \geq 0$ with $\nu_{i+1} = \nu_i + d_{i+1}$ for all $0 \leq i < k$ and $\nu = \nu_0$; we further say that $\sigma$ *reaches* valuation $\nu'$, if $\nu' = \nu_k$. We will need the following relationship between execution and traces: in case a pre-path $\sigma$ is actually a path, $\sigma$ can be executed from valuation $\nu$, if and only if there is a trace with initial valuation $\nu$ that uses the same sequence of transitions as $\sigma$. Two pre-paths $\sigma = t_1 \cdots t_k$ and $\sigma' = t'_1 \cdots t'_l$ can be *shuffled* into a pre-path $\sigma'' = t''_1 \cdots t''_{k+l}$, if $\sigma''$ is an order-preserving interleaving of $\sigma$ and $\sigma'$; formally, there are injective monotone functions $f : [1,k] \to [1,k+l]$ and $g : [1,l] \to [1,k+l]$ with $f([1,k]) \cap g([1,l]) = \emptyset$ such that $t''_{f(i)} = t_i$ for all $i \in [1,k]$ and $t''_{g(i)} = t'_i$ for all $i \in [1,l]$. Further, for $d \geq 1$ and pre-path $\sigma$, we denote by $\sigma^d = \underbrace{\sigma\sigma\cdots\sigma}_{d}$ the pre-path that consists of $d$ subsequent copies of $\sigma$.

For the remainder of this section, we fix a VASS $\mathcal{V}$ for which Algorithm 1 does not report exponential complexity and we fix the computed tree $T$ and bounds $\texttt{vExp}$, $\texttt{tExp}$. We further need to use the solutions to constraint system $(I)$ computed during the run of Algorithm 1: For every layer $l \geq 1$ and node $\eta \in \texttt{layer}(l)$, we fix a cycle $C(\eta)$ that contains $\mu(t)$ instances of every $t \in \mathit{Trns}(\texttt{VASS}(\eta))$, where $\mu$ is an optimal solution to constraint system $(I)$ during loop iteration $l$. The existence of such cycles is stated in Lemma 18 below. We note that this definition ensures $\mathit{val}(C(\eta)) = \sum_{t \in \mathit{Trns}(\texttt{VASS}(\eta))} D(t) \cdot \mu(t)$. Further, for the root node $\iota$, we fix an arbitrary cycle $C(\iota)$ that uses all transitions of $\mathcal{V}$ at least once.

**Lemma 18.** *Let $\mu$ be an optimal solution to constraint system $(I)$ during loop iteration $l$ of Algorithm 1. Then there is a cycle $C(\eta)$ for every $\eta \in \texttt{layer}(l)$ that contains exactly $\mu(t)$ instances of every transition $t \in \mathit{Trns}(\texttt{VASS}(\eta))$.*

*Proof Outline of the Lower Bound Theorem.*
**Step I)** We define a pre-path $\tau_l$, for every $l \geq 1$, with the following properties:

1) $\texttt{instance}(\tau_l, t) \geq N^{l+1}$ for all transitions $t \in \bigcup_{\eta \in \texttt{layer}(l)} \mathit{Trns}(\texttt{VASS}(\eta))$.
2) $\mathit{val}(\tau_l) = N^{l+1} \sum_{\eta \in \texttt{layer}(l)} \mathit{val}(C(\eta))$.
3) $\mathit{val}(\tau_l)(x) \geq 0$ for every $x \in \mathit{Var}$ with $\texttt{vExp}(x) \leq l$.
4) $\mathit{val}(\tau_l)(x) \geq N^{l+1}$ for every $x \in \mathit{Var}$ with $\texttt{vExp}(x) \geq l+1$.
5) $\tau_l$ is executable from some valuation $\nu$ with
   a) $\nu(x) \in O(N^{\texttt{vExp}(x)})$ for $x \in \mathit{Var}$ with $\texttt{vExp}(x) \leq l$, and
   b) $\nu(x) \in O(N^l)$ for $x \in \mathit{Var}$ with $\texttt{vExp}(x) \geq l+1$.

The difficulty in the construction of the pre-paths $\tau_l$ lies in ensuring Property 5). The construction of the $\tau_l$ proceeds along the tree $T$ using that the cycles $C(\eta)$ have been obtained according to solutions of constraint system $(I)$.

**Step II)** It is now a direct consequence of Properties 3)-5) stated above that we can choose a sufficiently large $k > 0$ such that for every $l \geq 0$ the pre-path $\rho_l = \tau_0^k \tau_1^k \cdots \tau_l^k$ (the concatenation of $k$ copies of each $\tau_i$, setting $\tau_0 = C(\iota)^N$), can be executed from some valuation $\nu$ and reaches a valuation $\nu'$ with

1) $\|\nu\| \in O(N)$,
2) $\nu'(x) \geq kN^{\texttt{vExp}(x)}$ for all $x \in \mathit{Var}$ with $\texttt{vExp}(x) \leq l$, and
3) $\nu'(x) \geq kN^{l+1}$ for all $x \in \mathit{Var}$ with $\texttt{vExp}(x) \geq l+1$.

The above stated properties for the pre-path $\rho_{l_{\max}}$, where $l_{\max}$ is the maximal layer of $T$, would be sufficient to conclude the lower bound proof except that we need to extend the proof from pre-paths to proper paths.

**Step III)** In order to extend the proof from pre-paths to paths we make use of the concept of shuffling. For all $l \geq 0$, we will define paths $\gamma_l$ that can be obtained by shuffling the pre-paths $\rho_0, \rho_1, \ldots, \rho_l$. The path $\gamma_{l_{\max}}$, where $l_{\max}$ is the maximal layer of $T$, then has the desired properties and allows to conclude the lower bound proof with the following result:

**Theorem 19.** *There are traces $\zeta_N$ with $\mathtt{init}(\zeta_N) \in O(N)$ such that $\zeta_N$ ends in configuration $(s_N, \nu_N)$ with $\nu_N(x) \geq N^{\mathtt{vExp}(x)}$ for all variables $x \in Var$ and we have $\mathtt{instance}(\zeta_N, t) \geq N^{\mathtt{tExp}(t)}$ for all transitions $t \in Trns(\mathcal{V})$.*

With Lemma 17 we get the desired lower bounds from Theorem 19:

**Corollary 20.** $\mathtt{vbound}_N(x) \in \Omega(N^{\mathtt{vExp}(x)})$ *for all $x \in Var$ and* $\mathtt{tbound}_N(t) \in \Omega(N^{\mathtt{tExp}(t)})$ *for all $t \in Trns(\mathcal{V})$.*

## 7 The Size of the Exponents

For the remainder of this section, we fix a VASS $\mathcal{V}$ for which Algorithm 1 does not report exponential complexity and we fix the computed tree $T$ and bounds vExp, tExp. Additionally, we fix a vector $z_l \in \mathbb{Z}^{St(\mathcal{V})}$ for every layer $l$ of $T$ and a vector $r_\eta \in \mathbb{Z}^{Var}$ for every node $\eta \in \mathtt{layer}(l)$ as follows: Let $r, z$ be an optimal solution to constraint system $(II)$ in iteration $l+1$ of Algorithm 1. We then set $z_l = z$. For every $\eta \in \mathtt{layer}(l)$ we define $r_\eta$ by setting $r_\eta(x) = r(x, \eta')$, where $\eta' \in \mathtt{layer}(l - \mathtt{vExp}(x))$ is the unique ancestor of $\eta$ in layer $l - \mathtt{vExp}(x)$. The following properties are immediate from the definition:

**Proposition 21.** *For every layer $l$ of $T$ and node $\eta \in \mathtt{layer}(l)$ we have:*

*1) $z_l \geq 0$ and $r_\eta \geq 0$.*
*2) $r_\eta^T d + z_l(s_2) - z_l(s_1) \leq 0$ for every transition $s_1 \xrightarrow{d} s_2 \in Trns(\mathtt{VASS}(\eta))$; moreover, the inequality is strict for all transitions $t$ with $\mathtt{tExp}(t) = l+1$.*
*3) Let $\eta' \in \mathtt{layer}(i)$ be a strict ancestor of $\eta$. Then, $r_{\eta'}^T d + z_i(s_2) - z_i(s_1) = 0$ for every transition $s_1 \xrightarrow{d} s_2 \in Trns(\mathtt{VASS}(\eta))$.*
*4) For every $x \in Var$ with $\mathtt{vExp}(x) = l+1$ we have $r_\eta(x) > 0$ and $r_\eta(x) = r_{\eta'}(x)$ for all $\eta' \in \mathtt{layer}(l)$.*
*5) For every $x \in Var$ with $\mathtt{vExp}(x) > l+1$ we have $r_\eta(x) = 0$.*
*6) For every $x \in Var$ with $\mathtt{vExp}(x) \leq l$ there is an ancestor $\eta' \in \mathtt{layer}(i)$ of $\eta$ such that $r_{\eta'}(x) > 0$ and $r_{\eta'}(x') = 0$ for all $x'$ with $\mathtt{vExp}(x') > \mathtt{vExp}(x)$.*

For a vector $r \in \mathbb{Z}^{Var}$, we define the *potential* of $r$ by setting $\mathtt{pot}(r) = \max\{\mathtt{vExp}(x) \mid x \in Var, r(x) \neq 0\}$, where we set $\max \emptyset = 0$. The motivation for this definition is that we have $r^T \nu \in O(N^{\mathtt{pot}(r)})$ for every valuation $\nu$ reachable by a trace $\zeta$ with $\mathtt{init}(\zeta) \leq N$ . We will now define the *potential* of a set of vectors $Z \subseteq \mathbb{Z}^{Var}$. Let $M$ be a matrix whose columns are the vectors of $Z$ and whose rows are ordered according to the variable bounds, i.e., if the row associated to variable $x'$ is above the row associated to variable $x$, then we have

$\mathtt{vExp}(x') \geq \mathtt{vExp}(x)$. Let $L$ be some lower triangular matrix obtained from $M$ by elementary column operations. We now define $\mathtt{pot}(Z) = \sum_{\text{column } r \text{ of } L} \mathtt{pot}(r)$, where we set $\sum \emptyset = 0$. We note that $\mathtt{pot}(Z)$ is well-defined, because the value $\mathtt{pot}(Z)$ does not depend on the choice of $M$ and $L$.

We next state an upper bound on potentials. Let $l \geq 0$ and let $B_l = \{\mathtt{vExp}(x) \mid x \in \mathit{Var}, \mathtt{vExp}(x) < l\}$ be the set of variable bounds below $l$. We set $\mathtt{varsum}(l) = 1$, for $B_l = \emptyset$, and $\mathtt{varsum}(l) = \sum B_l$, otherwise. The following statement is a direct consequence of the definitions:

**Proposition 22.** *Let $Z \subseteq \mathbb{Z}^{\mathit{Var}}$ be a set of vectors such that $r(x) = 0$ for all $r \in Z$ and $x \in \mathit{Var}$ with $\mathtt{vExp}(x) > l$. Then, we have $\mathtt{pot}(Z) \leq \mathtt{varsum}(l + 1)$.*

We define $\mathtt{pot}(\eta) = \mathtt{pot}(\{r_{\eta'} \mid \eta' \text{ is a strict ancestor of } \eta\})$ as the *potential* of a node $\eta$. We note that $\mathtt{pot}(\eta) \leq \mathtt{varsum}(l + 1)$ for every node $\eta \in \mathtt{layer}(l)$ by Proposition 22. Now, we are able to state the main results of this section:

**Lemma 23.** *Let $\eta$ be a node in $T$. Then, every trace $\zeta$ with $\mathtt{init}(\zeta) \leq N$ enters $\mathtt{VASS}(\eta)$ at most $O(N^{\mathtt{pot}(\eta)})$ times, i.e., $\zeta$ contains at most $O(N^{\mathtt{pot}(\eta)})$ transitions $s \xrightarrow{d} s'$ with $s \notin \mathit{St}(\mathtt{VASS}(\eta))$ and $s' \in \mathit{St}(\mathtt{VASS}(\eta))$.*

**Lemma 24.** *For every layer $l$, we have that $\mathtt{vExp}(x) = l$ resp. $\mathtt{tExp}(t) = l$ implies $\mathtt{vExp}(x) \leq \mathtt{varsum}(l)$ resp. $\mathtt{tExp}(t) \leq \mathtt{varsum}(l)$.*

The next result follows from Lemma 24 only by arithmetic manipulations and induction on $l$:

**Lemma 25.** *Let $l$ be some layer. Let $k$ be the number of variables $x \in \mathit{Var}$ with $\mathtt{vExp}(x) < l$. Then, $\mathtt{varsum}(l) \leq 2^k$.*

Theorem 11 is then a direct consequence of Lemma 24 and 25 (using $k \leq |\mathit{Var}|$).

## 8 Exponential Witness

The following lemma from [15] states a condition that is sufficient for a VASS to have exponential complexity[2]. We will use this lemma to prove Theorem 9:

**Lemma 26 (Lemma 10 of [15]).** *Let $\mathcal{V}$ be a connected VASS, let $U, W$ be a partitioning of Var and let $C_1, \ldots, C_m$ be cycles such that a) $val(C_i)(x) \geq 0$ for all $x \in U$ and $1 \leq i \leq m$, and b) $\sum_i val(C_i)(x) \geq 1$ for all $x \in W$. Then, there is a $c > 1$ and paths $\pi_N$ such that 1) $\pi_N$ can be executed from initial valuation $N \cdot \mathbf{1}$, 2) $\pi_N$ reaches a valuation $\nu$ with $\nu(x) \geq c^N$ for all $x \in W$ and 3) $(C_i)^{c^N}$ is a sub-path of $\pi_N$ for each $1 \leq i \leq m$.*

We now outline the proof of Theorem 9: We assume that Algorithm 1 returned "$\mathcal{V}$ has at least exponential complexity" in loop iteration $l$. According to Lemma 18, there are cycles $C(\eta)$, for every node $\eta \in \mathtt{layer}(l)$, that contain $\mu(t)$ instances of every transition $t \in \mathit{Trns}(\mathtt{VASS}(\eta))$. One can then show that the cycles $C(\eta)$ and the sets $U = \{x \in \mathit{Var} \mid \mathtt{vExp}(x) \leq l\}$, $W = \{x \in \mathit{Var} \mid \mathtt{vExp}(x) > l\}$ satisfy the requirements of Lemma 26, which establishes Theorem 9.

[2] Our formalization differs from[15], but it is easy to verify that our conditions a) and b) are equivalent to the conditions on the cycles in the 'iteration schemes' of [15].
